# Self-esteem and psychological immune competence of young people raised in child protection care

**DOI:** 10.3389/fpubh.2026.1700903

**Published:** 2026-05-08

**Authors:** Ildikó Erdei, Karolina Eszter Kovács, Erzsébet Rákó

**Affiliations:** 1 Doctoral Program on Educational Sciences, Doctoral School of Humanities, University of Debrecen, Debrecen, Hungary; 2 Institute of Psychology, Faculty of Arts, University of Debrecen, Debrecen, Hungary; 3 Department of Social Pedagogy, Faculty of Early Childhood Education and Special Educational Needs, University of Debrecen, Debrecen, Hungary

**Keywords:** child protection care, foster care, health behaviour, psychological immune competence, residential care, self-esteem

## Abstract

The psychological well-being of young people in child protection care may be significantly influenced by the type of care and the characteristics of the caregiving environment. The present study aimed to examine self-esteem, psychological immune competence, and health behaviour among 15–25-year-old youth living in foster families or residential care homes. The research was conducted through a questionnaire-based data collection at the child protection center of Hajdú-Bihar County. Respondents completed the Rosenberg Self-Esteem Scale and the junior version of the Psychological Immune Competence Inventory developed by Attila Oláh. The aim of the study was to explore how different forms of care are associated with specific psychological and health-related behavioural characteristics, and to identify potential correlations between self-esteem, psychological protective factors, and various dimensions of health behaviour. The study examined the self-esteem, psychological resilience, and health behaviours of adolescents and young adults raised in child protection care, comparing foster care and residential care settings. The sample consisted of 118 participants (ages 15–25; group home: *N* = 61; foster care: *N* = 57). Self-esteem was measured using the Rosenberg Self-Esteem Scale, psychological resilience using the Junior Psychological Resilience Questionnaire, and health behaviours were recorded via a questionnaire. For the analysis, we used the Mann–Whitney U test and Spearman’s correlation, adjusting for group imbalance through weighting. No significant gender differences were found in self-esteem or in the sub-dimensions of psychological immunocompetence. Foster care was associated with a more favourable pattern for certain components of immunocompetence and health behaviour indicators, whereas there was no difference in global self-assessment. The number of care placements was negatively associated with psychological resilience, whilst the duration of care was positively associated with it. Alcohol consumption showed a positive, presumably maladaptive, association with self-assessment. Our findings highlight the importance of the form and stability of care, as well as the need to support individual development within the child welfare system.

## 1 Introduction

The number of children in child protection care in Hungary is steadily increasing: in 2020, 22,934 and in 2024, 23,473 children and young adults received such care. Most come from low socio-cultural backgrounds, and their lives are shaped by loss, family trauma, poverty and separation. Entering the system represents a heavy psychological burden, lowering self-esteem and social support, and raising the risk of exclusion (1). Preserving mental health is therefore crucial, as it determines adaptability, coping and relationships (2).

Children removed from their families often grow up in non-ideal environments, so it is essential to examine how foster care and residential care influence their psychological outcomes. This study explores differences in self-esteem and psychological immunity among 15–25-year-olds living in foster families and residential homes. The research was conducted in Hajdú-Bihar County, where the proportion of children in specialised care is above the national average (64). Data collection took place in November 2023. The study aims to support the development of child protection care and highlight the importance of psychological support in promoting equal opportunities.

Specialised care becomes necessary when children face problems that cannot be resolved within the family. It provides temporary placement, sometimes with restrictions on parental rights (3), and covers the full range of services from temporary care to aftercare (4). Forms include children’s homes, residential homes and foster families, with removal only if risk cannot be managed in basic care (5). Reform schools also belong to the system (4).

Based on the distribution by age group, the largest proportion of children in child protection care system is in the 6–13 age group, followed by those aged 14–17, whilst the proportion of the youngest age groups is lower. This suggests that placement in child protection care becomes particularly prominent during the early school-age and adolescent stages of life. The lower proportion of younger age groups can be partly explained by the practise of adoption, as adoptive parents primarily prefer younger children (68). This selective nature is also relevant from a psychological perspective, as long-term placement at an older age and the lack of early stable attachments can create less favourable conditions for self-esteem and psychological adjustment (Schofield and Beek, 2008).

In 2024, 23,473 children and young adults were in care, including 21,212 minors. Whilst the number of 0–18-year-olds in Hungary declined between 2010 and 2024, the number in care increased, with two slight declines (2019–2020 and 2024). These may relate to COVID-19 restrictions and new legal requirements for foster parents. Between 2014 and 2021, the number in foster care rose from 14,205 to 16,158, whilst those in homes decreased; in 2023, the trend reversed (15,922 in foster care vs. 7,293 in homes) (6). Declining foster capacity is linked to ageing, economic difficulties and low financial recognition.

Removal usually results from low social status and financial hardship (7). Risk occurs when development is endangered by harmful conditions (4). Most children come from violent environments, which increases aggression and behavioural disorders (8). Separation itself is traumatic, often causing loss of self-esteem, insecurity and identity confusion. Children may show ambivalence in foster or residential care, with extremes in behaviour (9). Abuse before removal further intensifies aggression (10). The most vulnerable are adolescents moved from foster care to institutions, often with behavioural, learning, or integration problems, addictions or criminal tendencies (11, 70).

Young people raised in child protection care often face early-life trauma, neglect, and unstable family backgrounds, which significantly influence their psychological development, self-esteem, and coping mechanisms (12, 13). International research indicates that the quality of caregiving experiences, the stability of attachment relationships, and emotional connections with significant others fundamentally shape identity development and self-esteem during adolescence and young adulthood. Different forms of care provide varying psychosocial environments, which can lead to different developmental outcomes. Foster care generally offers a more stable, personal, and emotionally supportive environment, which can facilitate the formation of secure attachments and the development of a positive self-image. In contrast, residential care may be characterised by greater care instability, staff turnover, and the impersonality inherent in institutional settings, which can limit the formation of deeper relationships (14–17). Several key psychosocial mechanisms can be hypothesised to underlie these differences: the quality and stability of attachment relationships, the availability of emotional support, the predictability of the environment, and the degree of individual attention and reinforcement. These factors can directly influence the development of self-esteem and the functioning of psychological immunocompetence.

The type of care environment may influence psychological development through several key psychosocial mechanisms. Foster care typically provides a more stable, family-like environment characterised by consistent attachment figures, individualised attention, and emotional support. In contrast, residential care settings often involve rotating caregivers, larger group sizes, and less personalised interactions, which may limit the development of secure attachment and stable interpersonal relationships (16, 17). These differences are particularly important from a developmental perspective, as attachment security, perceived social support, and environmental stability are strongly associated with the formation of self-esteem and the development of adaptive coping capacities (18). Previous research also highlights that the loss of caregiver figures, disruptions in attachment relationships, and difficulties in identity formation play a central role in shaping adolescents’ self-esteem, particularly in vulnerable populations such as those in child protection care (19). Self-esteem is both a skill and an attitude, reflecting the gap between real and ideal self, influencing emotional stability, well-being and integration (20). Low self-esteem is linked to depression and loneliness, whilst excessive self-esteem can distort self-knowledge (21, 22). Childhood experiences and attachment strongly shape self-esteem, with positive feedback supporting self-acceptance (23, 24). Self-esteem and psychological immune competence are closely interrelated protective factors that play a decisive role in the development of resilience and mental well-being (25, 26). The psychological immune system is a set of cognitive, emotional, and motivational resources that promotes adaptive coping and emotional regulation in stressful situations. These internal resources are also linked to health behaviours: individuals with higher self-esteem and greater psychological immune competence are more likely to exhibit adaptive health behaviours, whereas risk behaviours may be more common among those with lower self-esteem and lower psychological immune competence. It can therefore be assumed that the type of care indirectly influences health behaviour through psychological resources.

From a conceptual perspective, these psychological resources may also be linked to health-related behaviours. Individuals with higher self-esteem and stronger psychological immune competence are more likely to engage in adaptive health behaviours and less likely to adopt risk behaviours, as they possess better self-regulation, future orientation, and coping strategies (27, 28). Accordingly, the present study assumes that the type of care may indirectly influence health behaviour through its impact on internal psychological resources. Children in child protection care are especially vulnerable: three-quarters were abused or neglected by their parents. This can lead to aggression and behavioural problems (29, 30). Foster parents and professionals play a key role in strengthening positive self-esteem. The psychological immune system (PI) is a cognitive toolkit that protects against internal and external threats, ensuring health and well-being (31, 32). Its cognitive, emotional and motivational subsystems provide flexible functioning and support self-development.

PI and self-esteem jointly contribute to adaptive functioning and effective coping, and may also be indirectly reflected in patterns of health behaviour. Psychological immune competence and self-esteem are closely related protective psychological resources that influence adaptation and coping. These internal resources may also be reflected in health-related behaviours, as individuals with stronger psychological resilience and higher self-esteem are more likely to engage in adaptive health behaviours and avoid risk behaviours.

The PI enables individuals to tolerate stress and activate coping, thus improving resilience (31, 32). Weak competence leads to dysfunction, whilst strong PI is associated with health. Children in care often face chronic stress, leading to psychological and behavioural problems (33, 34). Effective coping requires complex thinking and emotion regulation skills (9). Coping strategies – problem- or emotion-focused – strongly influence health and behaviour (35). Positive psychology highlights the role of strengths in developing coping skills (36). The PI also affects health behaviour and quality of life (37). Resilience is crucial for disadvantaged youth, including those in care (38–42). Strengthening the PI and coping strategies is essential for improving well-being.

### 1.1 Objectives, hypotheses

Although previous research has examined psychological outcomes of young people in child protection care, relatively few studies have simultaneously investigated self-esteem, psychological immune competence, and health-related behaviours within a unified framework, particularly in the context of comparing foster care and residential care settings. Moreover, the mechanisms through which different care environments shape these psychological resources remain insufficiently clarified. The research aims to conduct a psychological study of young people in specialised child protection care, with a particular focus on comparing the self-esteem and psychological immune competence of those living in residential homes and with foster parents. The primary aim of this study is to examine differences in self-esteem and psychological immune competence between young people living in foster care and residential care. A secondary aim is to explore how these psychological factors are associated with selected health-related behaviours. The question is whether there is a significant difference between the two forms of care and what differences can be observed in the various forms of upbringing. The research is being conducted at the child protection centre in Hajdú-Bihar county, as the number of children receiving specialised child protection care is significant here (43). To ensure conceptual clarity, the research questions were structured to distinguish between primary psychological variables and secondary exploratory outcomes. The primary research question of the study was whether there are significant differences in self-esteem and psychological immune competence between young people living in foster care and those in residential care. In addition, as a secondary aim, the study explored how these psychological factors are related to selected health-related behaviours, as well as how they are associated with age, length of care, and the number of care settings. The formulation of the hypotheses is based on the psychosocial mechanisms described in the literature, which suggest that a more stable and emotionally supportive caregiving environment may lead to more favourable psychological outcomes.

The following questions arose during the research:

1) Are there demonstrable sex differences in psychological immune competence and self-esteem in the special sample?
2) What differences are there between the types of care in these variables?
3) Is there a significant relationship between psychological immune competence, self-esteem, age, length of care and number of care settings?

The research was conducted at a child protection centre in Debrecen, where, after obtaining the necessary ethical approvals, we distributed questionnaires to children and young adults. The data was returned online, and the questionnaires were processed by strict data protection regulations.

In line with the research questions, the following hypotheses were formulated:

- *H1*: No significant differences are expected based on biological sex in the dimensions of self-esteem and psychological immune competence.
- *H2*: Youth in foster care exhibit more favourable patterns of psychological immunocompetence and health behaviours than those in residential care, whilst differences in self-esteem are less clear-cut.
- *H3*: Age, duration of care, and the number of care settings are associated with the dimensions of psychological immune competence; however, the direction of these associations is not consistent.
- *H4*: Self-esteem shows a significant association with health behaviours, particularly with regard to risk behaviours.

## 2 Materials and methods

### 2.1 Sample

The target group of the study is divided into two subgroups: young people living in residential homes and those raised by foster families. Both groups consisted of young people aged 15–25 born between 1998 and 2008. The age range of 15–25 years was selected to reflect the structure of the child protection system, where young people may remain in care beyond the age of 18 within aftercare provision. Although this range includes both adolescents and young adults, they share similar institutional contexts and developmental challenges related to care experience. Nevertheless, this heterogeneity is acknowledged as a limitation of the study. Inclusion criteria were: (1) being between 15 and 25 years of age, and (2) currently living in foster care or residential care in Hajdú-Bihar County. Participation was voluntary, and respondents were recruited through child protection professionals (residential care workers and foster parent counsellors), who distributed the questionnaire link to eligible participants. Only fully completed questionnaires were included in the final analysis. Incomplete responses were excluded.

The sample consisted of 118 participants aged 15–25 (mean 19.7; SD = 2.9); the participants came from two groups: residential care homes (*N* = 61) and foster care (*N* = 57). Sampling was conducted via a gatekeeper with voluntary responses; this was a non-probability sample, which we corrected for population imbalances between groups using weighting. At the same time, the aim of the study was to conduct a comprehensive survey of the available target population. To this end, a professional working at the child protection centre (gatekeeper) was tasked with distributing the questionnaire link to all affected youth who were accessible during the study period. We sent the questionnaire to a total of 137 individuals who were accessible at the child protection centre during that period. Of the 118 responses received, all were evaluable, as we set every question in the online questionnaire as mandatory, so there were no incomplete responses. Non-respondents either did not wish to complete the questionnaire or were not at home due to unauthorised absence and were not available online during the completion period. The questionnaire was thus accessible to all potential participants, ensuring an effort to reach the entire population within the institutional setting under study. Participation was anonymous and voluntary, which complies with ethical requirements but also carries the potential for self-selection bias. The inclusion and exclusion criteria, the number of individuals contacted, the response rate, and the handling of missing data were documented in detail.

### 2.2 Procedure

The research was conducted using online data collection, and convenience sampling was applieds. Informed consent was obtained from all participants prior to participation. In the case of participants under the age of 18, informed consent was also obtained from their legal guardians. Participation was voluntary and anonymous. The study was conducted in accordance with the Declaration of Helsinki and approved by the Ethics Committee of the Institute of Psychology at ELTE (approval number: 2023/410). All participants were informed about the purpose of the study, the voluntary nature of participation, and data confidentiality.

The link to the questionnaire was shared with the target group by the residential care workers and foster parent counsellors of the Child Protection Centre. Data collection took place in November 2023, and participation was anonymous and voluntary. During the research, respondents could interrupt the questionnaire at any time, and data management was carried out following the required ethical permits. The data was stored on Google Drive and in SPSS statistical databases and was not disclosed to third parties. Archiving took place after the research was completed.

The young people participating in the research completed a questionnaire package on the Google Forms platform, which included a sociodemographic questionnaire and two validated psychological tests. The sociodemographic questionnaire covered the age, educational background, years spent in child protection care and health behaviour of the respondents. To assess self-esteem, we used Rosenberg’s (71) Self-Esteem Scale, which measures global self-esteem and is one of the best-known and most reliable measurement tools. Psychological immune competence was assessed using the junior version of the questionnaire developed by Attila Oláh, which consists of 48 questions and contains 16 scales for measuring personal protective traits. Both questionnaires are reliable, with Cronbach’s alpha values of ≥ 0.857 for the Rosenberg scale and ≥0.7–0.9 for the PIK.

- Rosenberg Self-Esteem Scale (RSES): measures global self-esteem; 10 items; 4-point Likert scale. Reliability: Cronbach’s *α* = 0.86 in the present sample.
- Psychological Immune Competence Questionnaire (PIK) Junior Version: 24 items, 4-point Likert scale. The model consists of three interacting subsystems (approach-monitoring, mobilising-executive, and self-regulatory systems), which together ensure adaptive functioning and the continuity of self-development. The Junior Psychological Immune Competence Questionnaire (PsICJ) used in the present study measures the following, consolidated dimensions (Cronbach’s α = 0.81–0.89):

◦ Sense of Control: the individual’s belief that the course of events in a given life situation in which they are involved depends largely on them.
◦ Sense of coherence: the ability to understand connections, a strong belief that things will unfold in a reasonable manner and that they are predictable.
◦ Positive thinking/Optimism: the tendency to anticipate favourable changes and positive outcomes.
◦ Synchronisation Ability: the ability to fully attune to the environment, as well as the abilities of attentiveness and conscious behavioural control.
◦ Emotional Control: the ability to manage negative emotions arising from failures and threats, and to channel them into constructive behaviour

- Health Behaviour Questionnaire: frequency of smoking, alcohol consumption, exercise, and diet.

### 2.3 Statistical analysis

Statistical calculations were performed using SPSS 23.0. The data were first subjected to normality testing using Kolmogorov–Smirnov and Shapiro–Wilk tests. Since the variables did not show a normal distribution, non-parametric methods were used: Mann–Whitney tests were used to compare groups, and Spearman’s rank correlation was used to test for associations.

Since the data did not follow a normal distribution, we used Mann–Whitney U tests to compare the groups and Spearman’s correlation to examine the relationships between variables. Due to differences in group sizes (group home *N* = 61; foster family *N* = 57), we applied weighting in the statistical analyses so that the results would proportionally reflect the population sample. Analyses would better reflect the population proportions.

We performed the weighting using the following formula:


Weight(i)=N(largest group)/N(i)

where *N (i)* is the sample size of the given group, *N (largest group)* is the sample size of the larger group. Thus, the weight for those living in group homes is 1.0, whilst for foster parents it is 1.0702.

## 3 Results

### 3.1 Differences in self-esteem and psychological immune competence along sex

Given the non-normal distribution, we used the Mann–Whitney test to test the results. The group averages are shown in Table 1, and the significance values are shown in Table A1.

**Table 1:** Sex differences in psychological variables (source: own compilation).

Variable	Girl	Boy
Self-regulating subsystem		
Emotional control	7.37	7.11
Irritability control	8.26	8.23
Impulse control	7.55	7.79
Synchronicity	7.77	8.17
Total	30.95	31.30
Approach-belief subsystem		
Self-efficacy	8.26	8.75
Social creating capacity	7.85	7.55
Social mobilising capacity	7.32	7.38
Social monitoring capacity	8.17	8.23
Goal orientation	7.43	7.91
Total	39.03	39.81
Monitoring-creating-executing subsystem		
Creative self-concept	7.28	7.43
Problem solving capacity	8.23	8.43
Change and challenge orientation	8.40	8.79
Sense of self-growth	8.78	9.08
Sense of control	8.57	8.83
Sense of coherence	7.89	7.75
Positive thinking	8.54	8.55
Total	57.69	58.87
Self-esteem	26.95	26.79
Self-assessed health awareness	6.43	6.91

No significant sex differences can be found in any of the variables. The differences are minimal in terms of the self-regulatory system, the mobilising-creative-executing system and the approaching-monitoring system, with a difference of approximately 1 point (Table A1). The same can be observed in terms of self-esteem and self-rated health awareness. The hypothesis that there are significant sex differences in psychological immune competence and self-esteem in favour of boys was not confirmed in any of the cases.

### 3.2 Differences in self-esteem, psychological immune competence and health behaviour along the type of care

To test the second hypothesis, we used the Mann–Whitney test, similar to the previous one. The group averages are shown in Table 1, and the significance values are shown in Table A2.

In the case of psychological immune competence, there are significant differences in the subsystems. Within each subsystem, there are significant differences in optimism, sense of coherence, sense of control, sense of growth, creative self-concept, goal orientation, social resource monitoring ability, social resource mobilisation ability, self-esteem, synchronicity, impulse control, irritability control and emotional control. There is no significant difference in self-esteem (*p* = 0.240).

We also examined the frequency of individual health behaviour variables among foster parents and young people living in residential care homes (Table A3). There is a significant difference in the distribution of smoking frequency (*p* = 0.005). Among those living in residential homes, never smokers are underrepresented, whilst among children living with foster parents, the number of daily smokers is overrepresented. The data in Table A5 illustrate this. According to domestic research, smoking is the most widespread addiction in children’s homes (44). According to staff and residents of child protection institutions, smoking is a widespread addiction among young people in care as a means of stress relief. Children start smoking at a very young age, between 10 and 13 years old. If they do not have enough money to buy cigarettes, they search for cigarette butts (45).

No significant difference in distribution was found between the groups in terms of alcohol consumption (*p* = 0.339), as shown in Table A4. Alcohol consumption is less common among those in specialised child protection care and is more associated with specific occasions (45). The study by Kaló et al. also seems to support this, finding that alcohol consumption is hardly characteristic of those in care at home (44).

There was also a significant difference in distribution about drug experimentation (*p* = 0.01), as shown in Table A5. Young people raised in residential homes were overrepresented among those who had already tried some kind of drug, whilst this was much less common among young people raised by foster parents. Domestic research also shows that young people’s exposure to drugs varies according to the type of care they receive (46, 47), which seems to support my research findings. One reason for this may be that foster parents have a closer relationship with the child, as they raise them in their own home. In contrast, in residential homes, up to twelve children live together (48), where four child supervisors and one residential home educator carry out the educational work. Foster care provides more opportunities for developing personal relationships and learning social skills than residential homes (49). This may explain why drug use is less common in foster care (50, 51), which is also true for smaller residential homes, but is more common among children raised in large children’s homes (46). However, due to their low price, the use of new psychoactive substances is widespread (44). In terms of drug use, children raised in children’s homes are much more affected than the average population of the same age group (52). Children in specialised child protection care are at significant risk of drug use, which is higher in their case than in the average population (53). A significant number of young people are removed from their families due to addiction problems, mainly drug addiction. This is often due to existing family patterns that children and young adults bring with them from their socio-cultural background. However, some young people first encounter drugs in foster homes (54).

A significant difference in distribution was also found in energy drink consumption (*p* = 0.001), as shown in Table A6. Among young people living with foster parents, those who never or rarely consume energy drinks were overrepresented. In contrast, among those living in residential homes, those who consume energy drinks several times a day were significantly overrepresented.

Finally, there is also a significant difference in the distribution of coffee consumption frequency (*p* < 0.001), as shown in Table A7. Among young people raised in their parents’ homes, those who never consume coffee are overrepresented. In contrast, the proportion of those who consume coffee once a month or once a week is significantly higher among young people raised by foster parents.

The second hypothesis was only partially supported. Although significant differences were found in several dimensions of psychological immune competence and in certain health-related behaviours, no significant difference was observed in global self-esteem. Therefore, the hypothesis was not fully confirmed.

### 3.3 Connection between psychological immune competence and age, number of care settings and length of care

First, we explored the relationships between age and psychological variables, using Spearman’s rank correlation method due to the non-normal distribution of the data. There is a moderate negative correlation between age and optimism (*r* = −0.213; *p* = 0.021), i.e., the older the child, the lower the level of optimism. There is also a moderate negative relationship with the sense of coherence, i.e., the older the child, the lower the level of coherence. In addition, with increasing age, the levels of perseverance and impulse control are also lower. In contrast, social resource monitoring ability increases with age, indicating a moderate positive correlation between the two variables. The same is true for social resource mobilisation ability, synchronisation ability and irritability control. All of these are illustrated in Table 2.

**Table 2:** Differences in psychological variables based on the type of care (source: own compilation).

Variables	Foster parents	Residential care home
Self-regulating system	34.07	28.34
Emotional control	7.82	6.72
Irritability control	8.81	7.72
Impulse control	9.04	6.38
Synchronisation ability	8.40	7.52
Mobilising creative executive system	41.28	37.61
Self-esteem	9.77	7.28
Social resource creation ability	7.86	7.57
Social resource mobilisation capacity	7.05	7.62
Social resource monitoring capacity	8.25	8.15
Perseverance	8.35	6.98
Approach monitoring system	62.49	54.23
Resource creation ability	7.02	7.66
Ability to mobilise resources	8.51	8.15
Ability to monitor resources	9.74	7.49
Sense of growth	9.84	8.05
Sense of control	9.89	7.56
Sense of coherence	8.05	7.62
Optimism	9.44	7.70
Rosenberg	26.65	29.10
Self-assessed health awareness	7.16	6.16

We then focused on the relationship between care time and psychological variables. The results show that care time is weakly positively correlated with control ability, the feeling of growth with resource monitoring ability, self-esteem, synchronisation ability, impulse control, irritability control, emotional control and, in terms of subsystems, with the mobilising executive subsystem and the self-regulating subsystem. No significant negative correlations were found.

Finally, we looked at the correlation between the number of care settings and psychological variables. The number of care settings shows a moderate negative correlation with optimism, control ability, sense of growth, resource monitoring ability, resource creation ability, social resource monitoring ability, synchronisation ability, impulse control and emotional control, and, in terms of subsystems, with the approach monitoring subsystem and the self-regulatory subsystem. No significant positive correlations were found.

It is likely that the longer a child spends in special care, the better they can adapt to their situation, thus showing more positive correlations with the length of care. A negative correlation can be observed with the number of care settings, as being ‘bounced around’ in the system has a negative impact on children and young adults, undermining their sense of security and surrounding them with unpredictability and uncertainty. A change in care setting means not only a change of residence, but in some cases also a change of town and school, which is difficult for individuals who already have difficulty adapting. According to Aczél (1991), young people who have lived with several foster parents and then been placed in a residential home or children’s home have the weakest personality. Foster parents mostly request a change in care when the child is in their teens because they see teenage problems as a failure in their parenting and feel disappointed in the child (11).

The third hypothesis was only partially supported. Whilst negative correlations were observed in several cases (e.g., with the number of care settings), the relationships with age were mixed, and length of care showed positive correlations with several psychological variables, which is not consistent with the original hypothesis.

## 4 Discussion

The examination of self-esteem and coping among students in specialised child protection care is particularly important in terms of reflecting on the specific challenges of the specialised care environment. Children and young people raised in such institutions often have backgrounds and experiences that pose numerous emotional and social challenges. Examining their self-esteem and coping skills allows professionals to accurately identify areas where support is needed and to plan tailored interventions.

Self-esteem plays an important role in a child’s development and well-being (26). Children in child protection care often face situations that can affect their self-esteem, such as family problems, trauma or neglect (13, 65, 69). Insecure attachment and emotional regulation difficulties resulting from abuse and neglect can reduce children’s self-esteem in the long term (12, 55). Research shows that low self-esteem among young people in child protection care may be associated with mental health problems such as depression and anxiety (56). Professionals should monitor these processes in order to provide support to those affected in developing their self-confidence and positive self-image. Examining coping strategies can help identify practical tools for dealing with life challenges, especially for those raised in the child protection system (25). Children growing up in specialised care institutions often encounter stressful situations and it is important that they acquire appropriate tools for problem solving, emotional regulation and adaptation. Developing such skills can contribute to their successful coping with difficulties and positive experiences in different areas of life (26).

In the first hypothesis, we examined the role of sex differences about children raised in specialised child protection care; however, no significant differences were found in this regard (57, 58). Differences in the health behaviour and coping of boys and girls raised in child protection care may depend on several factors, including individual background factors and the institutional environment. Both boys and girls respond similarly to the conditions typical of specialised care (Kirchner et al., 2010). This can be partly explained by the fact that they face similar backgrounds and difficulties, such as neglect or other traumas, which influence their behaviour and coping strategies regardless of sex (Kirchner et al., 2010; (57)). As a result, similar coping mechanisms and health behaviours may develop in both sexes, and the institutional environment also plays a role in this, as the same care and support are available to both sexes (57). Social and cultural variables may also influence the behaviour and coping strategies of boys and girls raised in child protection care; however, children raised in the same circumstances often respond similarly to life challenges, regardless of their sex (67).

In the second hypothesis, we sought to explore differences based on the type of care. The results suggest that young people raised in foster care show more favourable outcomes in several dimensions of psychological immune competence and certain health-related behaviours. OűHowever, overall, the findings point to a differentiated pattern: whilst foster care appears to be associated with more favourable outcomes in several domains of psychological functioning and certain health-related behaviours, this advantage is not universal. In particular, the absence of differences in global self-esteem suggests that not all aspects of psychological well-being are equally influenced by the type of care. The positive influence of foster parents can also be seen in other areas; for example, children who live with foster parents achieve significantly better academic results than their peers living in residential care homes (14). Children raised by foster parents generally grow up in a more favourable environment than their peers living in residential homes. Placement with foster parents offers numerous advantages for children’s emotional, social and mental well-being.

In our third hypothesis, I focused on the correlations between psychological variables and age, the number of care settings, and the length of care. In this case, the research results were ambivalent. Age showed mixed correlations with the psychological immune system, with positive correlations in some cases and negative, albeit not very strong but significant, correlations in others. However, the length of care showed a consistent picture, suggesting that a longer duration of care was associated with higher levels of certain psychological immune competence variables. In this case, however, it should be noted that the respondents were not separated according to the type of care, which may distort the results to some extent. In addition, a consistent research finding is that the number of care settings is negatively correlated with the functioning of the psychological immune system, which points to the fact that frequent changes in a child’s care setting can have a negative impact on their mental health.

It is important to consider that the observed differences between foster care and residential care may be influenced by several unexamined factors. These may include the severity of prior trauma, the reasons for placement, the stability of caregiving relationships, the duration and timing of placement, as well as differences in school support and broader social environments. In addition, selection processes may play a role, as children with more complex behavioural or psychological difficulties may be more likely to be placed in residential care settings. Therefore, the differences observed in this study cannot be attributed solely to the type of care. In addition, previous research suggests that psychological vulnerability in young people in care may not arise solely from the current care environment, but may also reflect earlier developmental experiences, including family trauma, disrupted attachment relationships, and fragile relational functioning prior to placement (59–63). This perspective further supports the interpretation that the associations observed in the present study are likely influenced by both pre-placement and in-care factors.

This study provides valuable insights, yet several limitations should be noted. The sample of 118 participants (residential homes: *N* = 61; foster care: *N* = 57) was statistically acceptable but did not reflect the actual distribution in child protection, where foster care is more prevalent. Thus, generalisation to the whole population should be made with caution. The research was also limited to Hajdú-Bihar County, a region with above-average child protection rates, but local socio-economic factors may differ from those elsewhere, reducing national applicability. Data collection relied on self-report questionnaires, raising the risk of misunderstanding or socially desirable responses, without qualitative methods to clarify answers. It should be noted that the sample may be subject to selection bias, as participation depended on accessibility and willingness to respond. Therefore, the results may not fully represent the entire population of young people in child protection care. Statistically, the non-normal distribution of variables required non-parametric tests, which, whilst appropriate, are less sensitive with small samples. Finally, the cross-sectional design captured only a single time point, preventing conclusions about causality or developmental change. In addition, the study relied on non-probability (convenience) sampling, which limits the representativeness of the findings. Participation was voluntary and conducted via an online questionnaire, which may have introduced self-selection bias, as individuals who chose to participate may differ systematically from those who did not. Due to the cross-sectional design, the findings reflect associations at a single point in time and do not allow for causal conclusions. Therefore, it cannot be determined whether the type of care influences psychological outcomes, or whether pre-existing differences between individuals contribute to the observed patterns. Another limitation is that the study did not control for potential confounding variables, such as the severity of prior trauma, reasons for placement, or differences in care trajectories, which may have influenced the observed associations. Finally, it should be noted that the junior version of the Psychological Immune Competence Inventory was used in a sample that included participants up to 25 years of age, which may limit the validity of the measurement for older respondents. The findings should therefore be interpreted as indicative of associations rather than causal effects.

## 5 Conclusion

The findings of this study suggest that the type of care is associated with several dimensions of psychological immune competence and certain health-related behaviours among young people in child protection care. However, this pattern is not consistent across all variables, as no significant differences were found in global self-esteem. In addition, the relationships with age and length of care proved to be more complex than initially hypothesised, showing mixed and in some cases unexpected patterns. Overall, the results point to a differentiated and non-uniform relationship between care context and psychological functioning, highlighting the importance of considering both individual and contextual factors in understanding the well-being of young people in child protection care.

The findings suggest several directions for future research. Longitudinal studies could track changes in self-esteem and psychological immunity over time, whilst qualitative methods (e.g., interviews, focus groups) may reveal young people’s lived experiences more deeply. Broader samples, including other counties or national data, would allow regional comparisons and more generalisable conclusions. Future work should also examine additional variables such as attachment, trauma, school performance, or social support, and consider the perspectives of educators as complementary sources. Finally, testing and evaluating intervention programmes—such as self-esteem or resilience training—would both enrich theory and strengthen psychological support in practise.

## Data Availability

The raw data supporting the conclusions of this article will be made available by the authors, without undue reservation.
