# Supplementary material for: Self-esteem and psychological immune competence of young people raised in child protection care
Source: Front Public Health. 2026 May 8;14:1700903. doi: 10.3389/fpubh.2026.1700903 (PMC13205669; doi:10.3389/fpubh.2026.1700903)
Supplement: Supplementary file 1 [file Table_1.docx]

**Table A1.** Results of statistical tests used to examine gender differences (Source: own compilation)

|  | Sig |
| --- | --- |
| Self-esteem | 0.675 |
| Positive Thinking | 0.972 |
| Sense of coherence | 0.455 |
| Sense of Control | 0.411 |
| Sense of Self-Growth | 0.352 |
| Change and Challenge Orientation | 0.229 |
| Problem Solving Capacity | 0.359 |
| Creative Self-Concept | 0.353 |
| Goal orientation | 0.070 |
| Social Monitoring Capacity | 0.778 |
| Social Mobilizing Capacity | 0.753 |
| Social Creating Capacity | 0.117 |
| Self-efficacy | 0.174 |
| Synchronicity | 0.120 |
| Impulse Control | 0.483 |
| Irritability Control | 0.891 |
| Emotional Control | 0 |
| **APPROACH-BELIEF SUBSYSTEM** | 0.316 |
| **MONITORING-CREATING-EXECUTING SUBSYSTEM** | 0 |
| **SELF-REGULATING SUBSYSTEM** | 0 |

**Table A2.** Results of statistical tests used to examine differences based on place of care (Source: own compilation)

|  | Sig |
| --- | --- |
| Self-esteem | 0.240 |
| Positive Thinking | 0.000 |
| Sense of coherence | 0.018 |
| Sense of Control | 0.000 |
| Sense of Self-Growth | 0 |
| Change and Challenge Orientation | 0 |
| Problem Solving Capacity | 0.100 |
| Creative Self-Concept | 0 |
| Goal orientation | 0 |
| Social Monitoring Capacity | 0.627 |
| Social Mobilizing Capacity | 0.001 |
| Social Creating Capacity | 0.133 |
| Self-efficacy | 0 |
| Synchronicity | 0 |
| Impulse Control | 0 |
| Irritability Control | 0 |
| Emotional Control | 0 |
| **APPROACH-BELIEF SUBSYSTEM** | 0.000 |
| **MONITORING-CREATING-EXECUTING SUBSYSTEM** | 0 |
| **SELF-REGULATING SUBSYSTEM** | 0 |

**Table A3.** Distribution of smoking frequency by place of care (Source: own compilation)

|  | | **Type of care** | | **Total** |
| --- | --- | --- | --- | --- |
|  |  | **institution** | **foster parent** |  |
| **Never** | N | 7 | 23 | 30 |
|  | Row % | **23.3** | **76.7** | 100 |
|  | Column % | 11.5 | 40.4 | 25.4 |
|  | Adjusted residual | - | 3 |  |
| **very rarely** | N | 3 | 2 | 5 |
|  | Row % | 60 | 4 | 100 |
|  | Column % | 4.9 | 3.5 | 4.2 |
|  | Adjusted residual | 0 | - |  |
| **monthly** | N | 0 | 1 | 1 |
|  | Row % | 0 | 1 | 100 |
|  | Column % | 0 | 1.8 | 0 |
|  | Adjusted residual | - | 1 |  |
| **weekly** | N | 2 | 2 | 4 |
|  | Row % | 50 | 50 | 100 |
|  | Column % | 3.3 | 3 | 3 |
|  | Adjusted residual | - | 0 |  |
| **daily** | N | 49 | 29 | 78 |
|  | Row % | **62.8** | **37** | 100.0 |
|  | Column % | 80.3 | 50.9 | 66.1 |
|  | Adjusted residual | 3 | - |  |
| **Total** | N | 61 | 57 | 118 |
|  | Row % | 51.7 | 48.3 | 100.0 |
|  | Column % | 100 | 100 | 100 |

Note: Values underlined in bold indicate significant differences. Adjusted residual values below -2 indicate underrepresentation, while values above 2 indicate overrepresentation.

**Table A4.** Distribution of alcohol consumption frequency by type of care (Source: own compilation)

|  | | **Type of care** | | **Total** |
| --- | --- | --- | --- | --- |
|  |  | **Home** | **foster parent** |  |
| **Never** | N | 6 | 7 | 13 |
|  | Row % | 46.2 | 53.8 | 100.0 |
|  | Column % | 9.8 | 12.3 | 11 |
|  | Adjusted residual | - | 0 |  |
| **very rarely** | N | 1 | 8 | 21 |
|  | Row % | 61.9 | 38.1 | 100.0 |
|  | Column % | 21.3 | 14.0 | 17. |
|  | Adjusted residual | 1 | -1 |  |
| **month** | N | 2 | 26 | 48 |
|  | Row % | 45.8 | 54.2 | 100.0 |
|  | Column % | 36.1 | 45.6 | 40.7 |
|  | Adjusted residual | - | 1 |  |
| **weekly** | N | 2 | 14 | 34 |
|  | Row % | 58.8 | 41.2 | 100.0 |
|  | Column % | 32.8 | 24.6 | 28.8 |
|  | Adjusted residual | 1 | - |  |
| **daily** | N | 0 | 2 | 2 |
|  | Row % | 0 | 10 | 100 |
|  | Column % | 0 | 3.5 | 1.7 |
|  | Adjusted residual | -1 | 1 |  |
| **Total** | N | 61 | 57 | 118 |
|  | Row % | 51.7 | 48.3 | 100.0 |
|  | Column % | 100 | 100 | 100 |

**Table A5.** Distribution of drug experimentation by place of care (Source: own compilation)

| **Drug experimentation** | | **Type of care** | | **Total** |
| --- | --- | --- | --- | --- |
|  |  | **Home** | **foster parents** |  |
| **No** | N | 32 | 43 | 75 |
|  | Row % | **42.7** | **57.3** | 100.0 |
|  | Column % | 52.5 | 75.4 | 63.6 |
|  | Adjusted residual | - | 2 |  |
| **Yes** | N | 2 | 14 | 43 |
|  | Row % | **67.4** | **32.6** | 100.0 |
|  | Column % | 47.5 | 24.6 | 36.4 |
|  | Adjusted residual | 2 | -2 |  |
| **Total** | N | 61 | 57 | 118 |
|  | Row % | 51.7 | 48.3 | 100.0 |
|  | Column % | 100 | 100 | 100 |

Note: Values underlined in bold indicate significant differences. Adjusted residual values below -2 indicate underrepresentation, while values above 2 indicate overrepresentation.

**Table A6.** Distribution of energy drink consumption by place of care (Source: own compilation)

| **Energy drink consumption** | | **Type of care** | | **Total** |
| --- | --- | --- | --- | --- |
|  |  | **Home** | **foster parent** |  |
| **never** | N | 1 | 9 | 10 |
|  | Row % | 10 | 90 | 100 |
|  | Column % | 1.6 | 15.8 | 8.5 |
|  | Adjusted residual | - | 2 |  |
| **less frequently** | N | 7 | 16 | 23 |
|  | Row % | 30.4 | 69.6 | 100.0 |
|  | Column % | 11.5 | 28.1 | 19.5 |
|  | Adjusted residual | - | 2 |  |
| **per month** | N | 2 | 7 | 9 |
|  | Row % | 22.2 | 77.8 | 100.0 |
|  | Column % | 3.3 | 12.3 | 7.6 |
|  | Adjusted residual | - | 1 |  |
| **once a week** | N | 6 | 3 | 9 |
|  | Row % | 66.7 | 33 | 100.0 |
|  | Column % | 9.8 | 5.3 | 7 |
|  | Adjusted residual | 0 | -0 |  |
| **weekly** | N | 1 | 6 | 16 |
|  | Row % | 62.5 | 37.5 | 100.0 |
|  | Column % | 16.4 | 10.5 | 13.6 |
|  | Adjusted residual | 0 | - |  |
| **once a day** | N | 2 | 11 | 31 |
|  | Row % | 64.5 | 35 | 100.0 |
|  | Column % | 32.8 | 19.3 | 26.3 |
|  | Adjusted residual | 1 | - |  |
| **Several times a day** | N | 1 | 5 | 20 |
|  | Row % | 75 | 25 | 100 |
|  | Column % | 24.6 | 8.8 | 16 |
|  | Adjusted residual | 2 | - |  |
| **Ttal** | N | 61 | 57 | 118 |
|  | Row % | 51.7 | 48.3 | 100 |
|  | Column % | 100 | 10 | 100 |

Note: Values underlined in bold indicate significant differences. Adjusted residual values below -2 indicate underrepresentation, while values above 2 indicate overrepresentation.

**Table A7.** Frequency of coffee consumption depending on the place of care (Source: own edition)

| **Coffee consumption** | | **Type of care** | | **Total** |
| --- | --- | --- | --- | --- |
|  |  | **Home** | **foster parent** |  |
| **never** | N | 27 | 12 | 39 |
|  | Row % | 69.2 | 30.8 | 100.0 |
|  | Column % | 44.3 | 21.1 | 33.1 |
|  | Adjusted residual | 2 | -2.7 |  |
| **less frequently** | N | 16 | 8 | 24 |
|  | Row % | 66.7 | 33 | 100.0 |
|  | Column % | 26.2 | 14 | 20.3 |
|  | Adjusted residual | 1 | - |  |
| **monthly** | N | 4 | 13 | 17 |
|  | Row % | 23.5 | 76.5 | 100.0 |
|  | Column % | 6.6 | 22.8 | 14.4 |
|  | Adjusted residual | - | 2 |  |
| **once a week** | N | 2 | 1 | 13 |
|  | Row % | 15.4 | 84.6 | 100.0 |
|  | Column % | 3.3 | 19.3 | 11 |
|  | Adjusted residual | - | 2 |  |
| **per week** | N | 5 | 5 | 10 |
|  | Row % | 50 | 5 | 100.0 |
|  | Column % | 8 | 8 | 8.5 |
|  | Adjusted residual | - | 0 |  |
| **once a day** | N | 3 | 8 | 11 |
|  | Row % | 27.3 | 72.7 | 100.0 |
|  | Column % | 4.9 | 14 | 9.3 |
|  | Adjusted residual | - | 1 |  |
| **several times a day** | N | 4 | 0 | 4 |
|  | Row % | 10 | 0 | 100 |
|  | Column % | 6.6 | 0 | 3 |
|  | Adjusted residual | 2 | - |  |
| **Total** | N | 61 | 57 | 118 |
|  | Row % | 51.7 | 48.3 | 100.0 |
|  | Column % | 100 | 100 | 100 |

Note: Values underlined in bold indicate significant differences. Adjusted residual values below -2 indicate underrepresentation, while values above 2 indicate overrepresentation.

**Table A8.** Correlation between age, length of care and number of care settings with individual psychological variables (Source: own compilation)

|  |  | **Age** | **Length of care** | **Number of care settings** |
| --- | --- | --- | --- | --- |
| **self-esteem** | r | -0.042 | -0.137 | 0 |
|  | p | 0 | 0 | 0.752 |
| **Positive Thinking** | r | -,213^*^ | 0.109 | -,349^**^ |
|  | p | 0 | 0 | 0 |
| **Sense of coherence** | r | -,265^**^ | 0 | -0.09 |
|  | p | 0.004 | 0.954 | 0.332 |
| **Sense of Control** | r | -0.117 | ,204^*^ | -,362^**^ |
|  | p | 0 | 0 | 0 |
| **Sense of Self-Growth** | r | 0.007 | ,212^*^ | -,246^**^ |
|  | p | 0.938 | 0 | 0.007 |
| **Change and Challenge Orientation** | r | -0.078 | ,219^*^ | -,325^**^ |
|  | p | 0 | 0 | 0 |
| **Problem Solving Capacity** | r | 0 | 0 | -0 |
|  | p | 0 | 0 | 0 |
| **Creative Self-Concept** | r | 0 | -0.089 | ,244^**^ |
|  | p | 0.153 | 0 | 0 |
| **MONITORING-CREATING-EXECUTING SUBSYSTEM** | r | -0 | ,188^*^ | -,330^**^ |
|  | p | 0 | 0 | 0 |
| **Goal orientation** | r | -,191^*^ | 0 | -,313^**^ |
|  | p | 0 | 0 | 0 |
| **Social Monitoring Capacity** | r | ,400^**^ | 0.052 | 0 |
|  | p | 0 | 0.579 | 0.485 |
| **Social Mobilizing Capacity** | r | ,289^**^ | 0 | 0 |
|  | p | 0 | 0.674 | 0.230 |
| **Social Creating Capacity** | r | 0 | 0 | - |
|  | p | 0 | 0.327 | 0 |
| **Self-efficacy** | r | -,207^*^ | ,187^*^ | -,325^**^ |
|  | p | 0.025 | 0.042 | 0 |
| **APPROACH-BELIEF SUBSYSTEM** | r | 0.01 | ,261^**^ | -,307^**^ |
|  | p | 0.845 | 0.004 | 0.001 |
| Synchronicity | r | ,316^**^ | ,346^**^ | -0.109 |
|  | p | 0 | 0 | 0 |
| **Implulse Control** | r | -,208^*^ | ,207^*^ | -,358^**^ |
|  | p | 0 | 0 | 0 |
| **Irritability control** | r | ,244^**^ | ,259^**^ | -0 |
|  | p | 0 | 0 | 0 |
| **Emotional control** | r | -0.120 | 0 | -,215^*^ |
|  | p | 0 | 0 | 0 |
| **SELF-REGULATING SUBSYSTEM** | r | 0.028 | ,333^**^ | -,291^**^ |
|  | p | 0.763 | 0 | 0 |

**Table A9.** Relationship between self-esteem and health behaviour indicators (Source: own compilation)

|  |  | **Self-assessment** |
| --- | --- | --- |
| **Frequency of smoking** | r | 0.127 |
|  | p | 0.169 |
| **Frequency of alcohol consumption** | r | ,234^*^ |
|  | p | 0 |
| **Frequency of drug use** | r | 0.046 |
|  | p | 0 |
| **Frequency of energy drink consumption** | r | 0.120 |
|  | p | 0.196 |
| **Frequency of coffee consumption** | r | -0.030 |
|  | p | 0.749 |
| **Self-rated health awareness** | r | -0.067 |
|  | p | 0 |
